# Supplementary material for: CRISPR-Cpf1 mediates efficient homology-directed repair and temperature-controlled genome editing
Source: Nat Commun. 2017 Dec 8;8:2024. doi: 10.1038/s41467-017-01836-2 (PMC5722943; doi:10.1038/s41467-017-01836-2)
Supplement: Supplementary file 1 — Supplementary Information [file 41467_2017_1836_MOESM1_ESM.pdf]

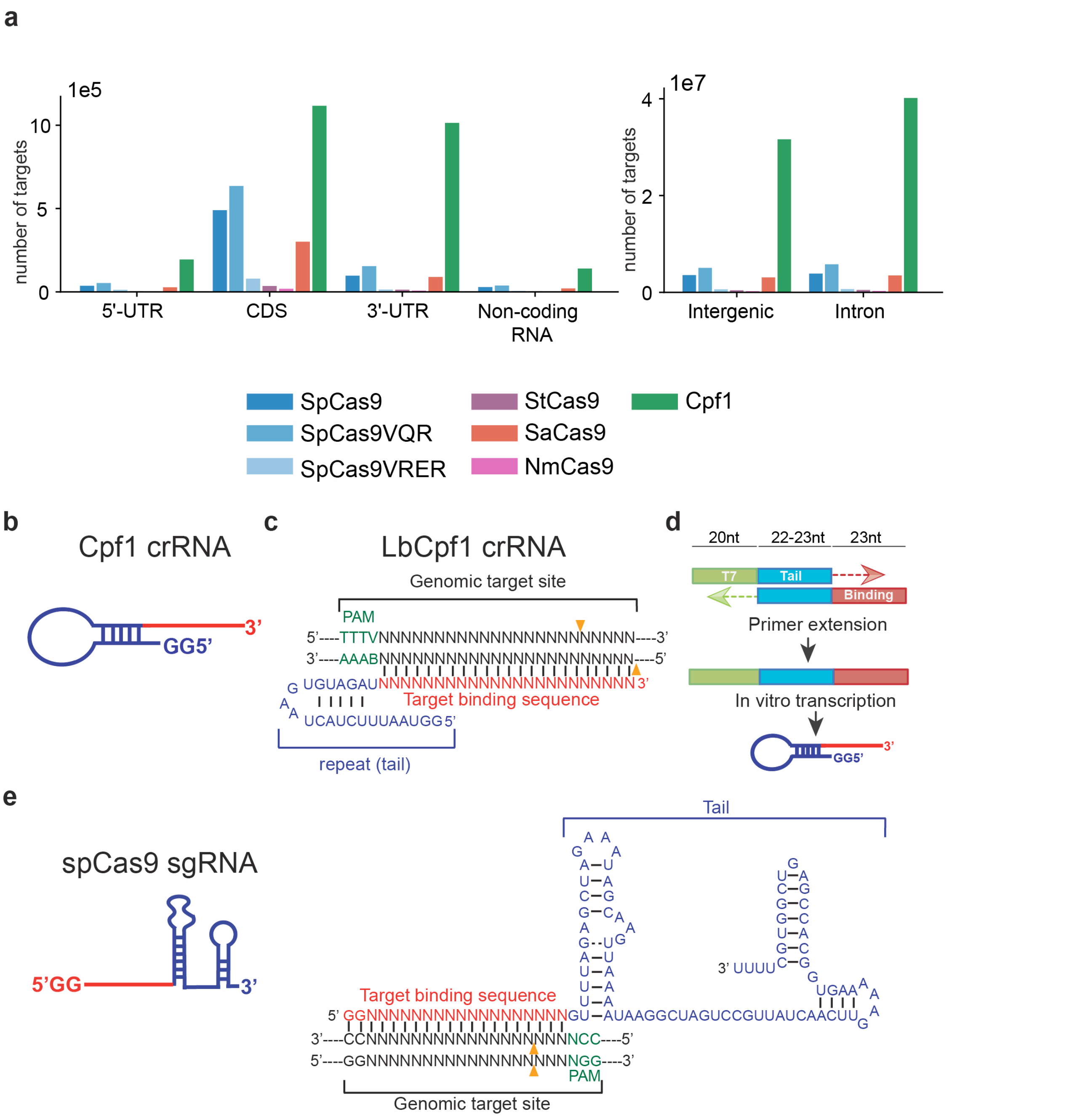

Supplementary Figure 1. CRISPR-Cpf1 system features.

a. Bar graphs showing number of targets of different CRISPR endonucleases in the zebrafish genome. Defined PAM sequences are available in Supplementary Data 1. SpCas9: *Streptococcus pyogenes* Cas9[1]; SpCas9VQR: *S. pyogenes* Cas9 VQR mutant[2]; SpCas9VRER: *S. pyogenes* Cas9 VRER mutant[2]; StCas9: *Streptococcus thermophilus* CRISPR 1 Cas9[3]; SaCas9: *Staphylococcus aureus* Cas9[4]; NmCas9: *Neisseria* meningiditis Cas9[3, 5]; Cpf1: *Acidaminococcus sp BV3L6 / Lachnospiraceae bacterium ND2006* Cpf1[6].

b. Schematic of crRNA structure.

c. Scheme showing a LbCpf1 crRNA (binding sequence in red, repeat in blue) binding to the genomic target site (black) and the PAM sequence 5'-TTTV (green). Orange triangles indicate predicted cleavage sites. (V=A,C,G; B=T,G,C).

d. PCR approach to obtain a 65bp (AsCpf1) or 66bp (LbCpf1) bp product used as template for crRNA *in vitro* transcription. An oligonucleotide containing the T7 promoter (green) followed by two Guanine and 20 (AsCpf1) or 21 nt (LbCpf1) of the invariable repeat (tail, in blue) for annealing is used in combination with oligonucleotide containing the reverse complement of the repeat and 23 nt of the binding sequence (in red).
e. Schematic of sgRNA structure (left) and scheme showing a sgRNA (binding sequence in red, tail in blue) binding to the genomic target site (black) and the PAM sequence 5'-NGG (green). Orange triangles indicate predicted cleavage sites. Adapted from Moreno-Mateos et al.[7] While sgRNA is ~100nt, crRNA is ~43nt, which facilitates *in vitro* synthesis.

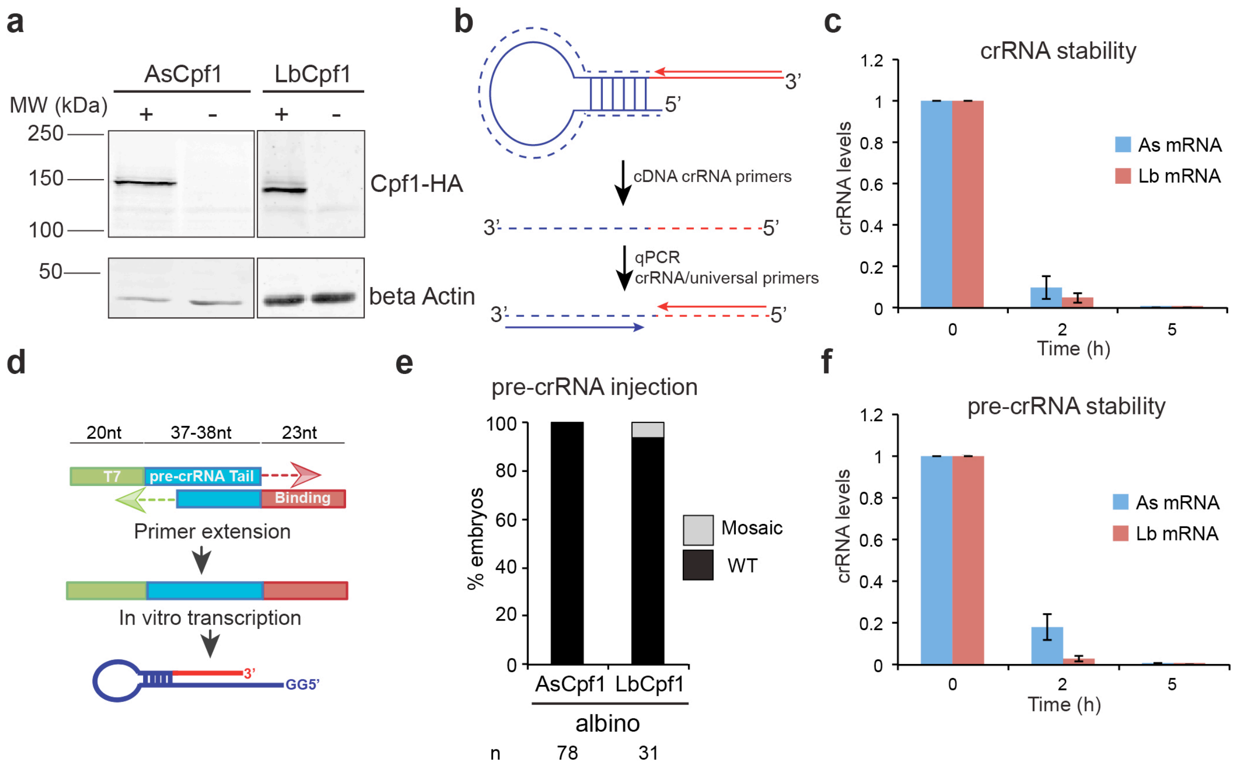

Supplementary Figure 2. crRNA are rapidly degraded in zebrafish.

a. Western blot showing Cpf1-HA protein in injected zebrafish embryos. Injected embryos (+) and uninjected (-) embryos as negative control were analyzed by using HA and beta-Actin antibodies. MW: Molecular weight marker (kilodalton).

b. Schematic illustrating crRNA-cDNA and qPCR strategy. Specific crRNA primers annealing to the target binding sequence (red) were used to make cDNA. qPCR was carried out using a universal primer annealing to the repeat region (blue) and the specific primers described above (Supplementary Data 1).

c. qRT–PCR analysis showing levels of crRNAs used for targeting *slc45a2* in Fig. 1c. Results are shown as the averages ± standard deviation of the mean for three crRNAs.

d. PCR approach to obtain a 80bp (AsCpf1) or 81bp (LbCpf1) product used as template for pre-crRNA *in vitro* transcription similar to that described in Supplementary Fig. 1d, except the oligonucleotide containing the T7 promoter (green) includes the 35 nt (AsCpf1) or 36 nt (LbCpf1) pre-crRNA repeat sequence (tail).

e. Phenotypic evaluation of pre-crRNAs (30 pg/pre-crRNA) and mRNA (100 pg) injections. Stacked barplots showing the percentage of mosaic (gray) and phenotypically WT (black) embryos 48 hpf after injection.

f. qRT–PCR analysis showing levels of crRNAs used for targeting *slc45a2* in Supplementary Fig. 2e. Results are shown as the averages ± standard deviation of the mean for three crRNAs.

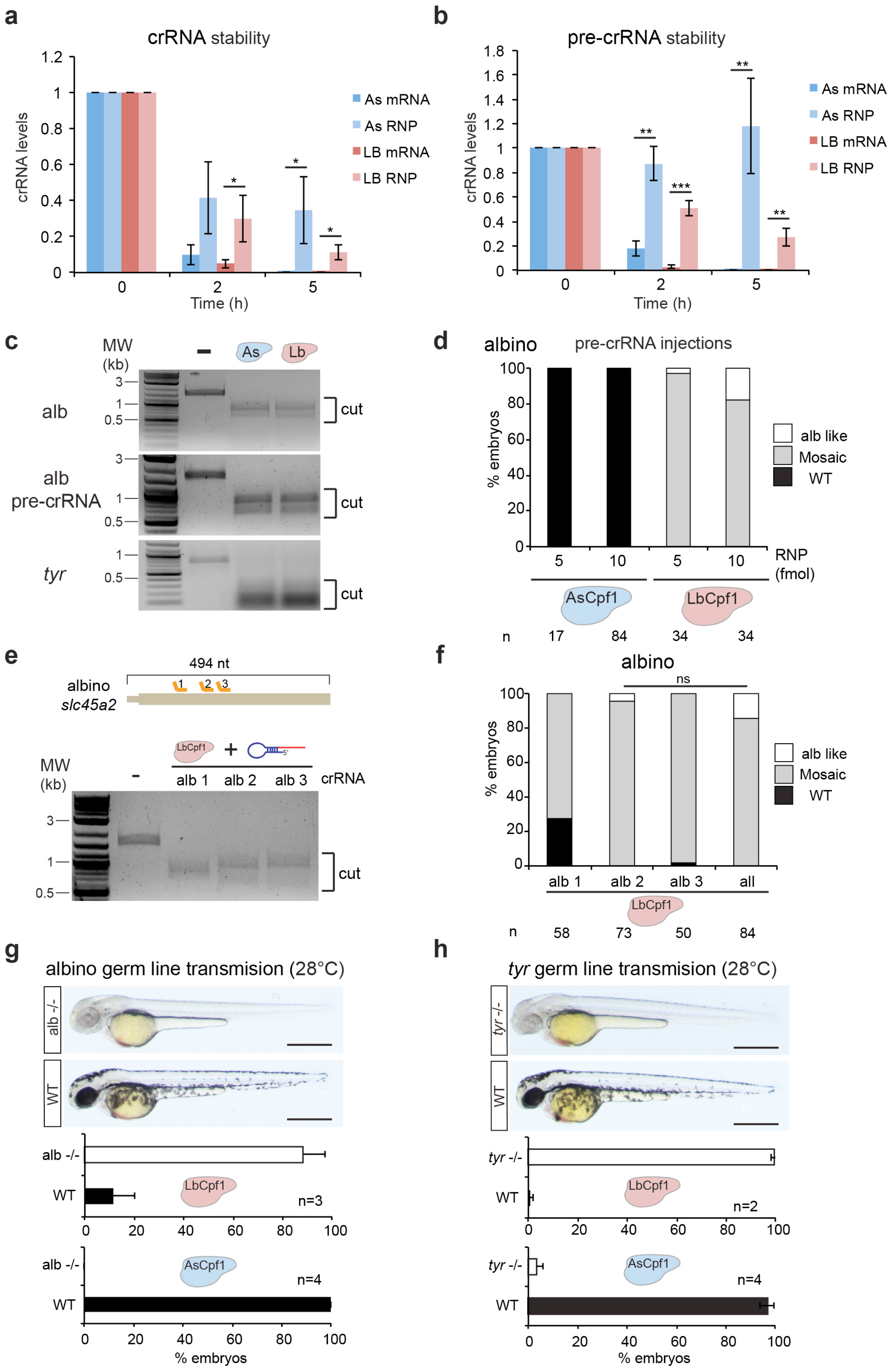
a
crRNA stability
crRNA levels
1.2
1
0.8
0.6
0.4
0.2
0
0
2
5
Time (h)
As mRNA
As RNP
LB mRNA
LB RNP
*
*
*
b
pre-crRNA stability
crRNA levels
1.8
1.6
1.4
1.2
1
0.8
0.6
0.4
0.2
0
0
2
5
Time (h)
As mRNA
As RNP
LB mRNA
LB RNP
**
***
**
**
c
MW
(kb)
-
As
Lb
alb
alb
pre-crRNA
tyr
3
1
0.5
cut
d
albino
pre-crRNA injections
% embryos
100
80
60
40
20
0
5
10
5
10
RNP
(fmol)
AsCpf1
LbCpf1
alb like
Mosaic
WT
n
17
84
34
34
e
494 nt
albino
slc45a2
1
2
3
LbCpf1
+
MW
(kb)
-
alb 1
alb 2
alb 3
crRNA
3
1
0.5
cut
f
albino
ns
% embryos
100
80
60
40
20
0
alb 1
alb 2
alb 3
all
LbCpf1
alb like
Mosaic
WT
n
58
73
50
84
g
albino germ line transmision (28°C)
alb -/-
WT
LbCpf1
n=3
0
20
40
60
80
100
AsCpf1
n=4
% embryos
h
tyr germ line transmision (28°C)
tyr -/-
WT
LbCpf1
n=2
AsCpf1
n=4
0
20
40
60
80
100
% embryos

Supplementary Figure 3. Cpf1-crRNA RNP complexes stabilize crRNA but only LbCpf1 allows robust genome editing in zebrafish.

a. qRT–PCR analysis showing levels of crRNAs used for targeting *slc45a2* in Fig. 1c and 1e (5 fmol). Results are shown as the averages ± standard deviation of the mean for three crRNAs. The data were subjected to two-tailed Student's t-test. (*) P< 0.05.

b. qRT–PCR analysis showing levels of crRNAs used for targeting *slc45a2* in Supplementary Fig. 2e and 3d (5 fmol). Results are shown as the averages ± standard deviation of the mean for three crRNAs. The data were subjected to two-tailed Student's t-test. (**) P < 0.01, (***) P < 0.001.

c. In vitro cleavage assay using 10 pmol of AsCpf1 RNP or LbCpf1 RNP complexes containing a mix of 3 crRNAs (Fig. 1a) and a ~1.6 Kb (~ 0.11 pmol) PCR product including crRNA target sites for *slc45a2* (alb) or 20 pmol of AsCpf1 RNP or LbCpf1 RNP complexes containing a mix of 3 crRNAs and a 0.77 Kb (~ 0.23 pmol*)* PCR product including crRNA target sites for *tyrosinase*. Incubations were carried out at 37°C for 90 min. MW: Molecular weight marker (kilobase).

d. Phenotypic evaluation of Cpf1-pre-crRNA RNP complexes injections targeting *slc45a2*. Stacked barplots showing the percentage of alb-like (white), mosaic (gray) and phenotypically WT (black) embryos 48 hpf after injection. Number of embryos evaluated (n) is shown for each condition.

e. *In vitro* cleavage assay using 10 pmol of LbCpf1 RNP complexes containing individual crRNAs and a ~1.6 Kb (~ 0.11 pmol) PCR product including crRNA target sites for *slc45a2*. Incubations were carried out at 37°C for 90 min. MW: Molecular weight marker (kilobase).

f. Phenotypic evaluation of individual Cpf1-crRNA RNP (10 fmol) complexes injections targeting *slc45a2*. Stacked barplots showing the percentage of alb-like (white), mosaic (gray) and phenotypically WT (black) embryos 48 hpf after injection. Number of embryos evaluated (n) is shown for each condition. No significant (ns) differences were found between the Cpf1 activity in the pool of 3 crRNAs (all) and the most efficient crRNA in the pool (alb 2) (χ^2 test).

g. Phenotypes obtained from F0 *slc45a2* (albino) mutants (48 hpf old embryos) in crosses (lateral views) using LbCpf1-injected fish (top panel) developed at 28°C. Scale bar, 0.5 mm. Percentage of alb -/- and WT obtained from independent (n) in crosses using either LbCpf1- (middle panel) or AsCpf1- (bottom panel) injected fish developed at 28°C. Results are shown as the averages ± standard deviation of the mean for different crosses (n).

h. Phenotypes obtained from F0 *tyr*osinase mutants (48 hpf old embryos) in crosses (lateral views) using LbCpf1-injected fish developed at 28°C (top panel). Scale bar, 0.5 mm. Percentage of *tyr* -/- and WT obtained from independent (n) in crosses using either LbCpf1- (middle panel) or AsCpf1- (bottom panel) injected fish developed at 28°C. Results are shown as the averages ± standard deviation of the mean for different crosses (n).

zebrafish *slc45a2*

#1

cr1:Δ15 cr2:Δ17

```
1                   GAGGACTGTTTAGACAACATGGAAGCG---------------GTGGAGCC
WT                  GAGGACTGTTTAGACAACATGGAAGCGGCTGTTTTTGGAGTGGTGGAGCC
                    ***************************               ********

1                   AGGCGCTCTAGGGGAAGGTTGATTATGCACGGGTCAGCCATGTTTGGAAG
WT                  AGGCGCTCTAGGGGAAGGTTGATTATGCACGGGTCGGCCATGTTTGGAAG
                    **************************************************

1                   GGAATTCT-----------------GCGTTTGTCACGCCAGTGTTGCTGA
WT                  GGAATTCTGCTACGCTGTTGAGGCTGCGTTTGTCACGCCAGTGTTGCTGA
                    ************             *************************
```

#2

cr1:Δ16

```
2                   GAGGACTGTTTAGACAACATGG----------------AGCGGTGGAGCC
WT                  GAGGACTGTTTAGACAACATGGAAGCGGCTGTTTTTGGAGCGGTGGAGCC
                    **********************                ************
```

#3

cr2:Δ16

```
3                   GCCATGTTTGGAAGGGAATTCT-----------------GCGTTTGTCAC
WT                  GCCATGTTTGGAAGGGAATTCTGCTACGCTGTTGAGGCTGCGTTTGTCAC
                    **********************                 ***********
```

#4

cr3:Δ11

```
4                   GAGGCTGCGTTTGTCACGCCAGTGTT-----------GACTTCCCAGACG
WT                  GAGGCTGCGTTTGTCACGCCAGTGTTGCTGAGCGTTGGACTTCCCAGACG
                    **************************           *************
```

#5

cr2:Δ9

```
5                   GCCATGTTTGGAAGGGAATTCTGCTACGC---------TGCGTTTGTCAC
WT                  GCCATGTTTGGAAGGGAATTCTGCTACGCTGTTGAGGCTGCGTTTGTCAC
                    *****************************         ************
```

#6

cr1:Δ16 cr2:Δ4

```
6                   GAGGACTGTTTAGACAACATGG----------------AGCGGTGGAGCC
WT                  GAGGACTGTTTAGACAACATGGAAGCGGCTGTTTTTGGAGTGGTGGAGCC
                    **********************                ************

6                   TCCAAGGCGCTCTAGGGGAAGGTTGATTATGCACGGGTCAGCCATGTTTG
WT                  TCCGAGGCGCTCTAGGGGAAGGTTGATTATGCACGGGTCGGCCATGTTTG
                    **************************************************
```

```
6                GAAGGGAATTCTGCTACGC----GAGGCTGCGTTTGTCACGCCAGTGTTG
WT               GAAGGGAATTCTGCTACGCTGTTGAGGCTGCGTTTGTCACGCCAGTGTTG
                 *******************    ***************************
```

#7

cr2:Δ11

```
7                GCCATGTTTGGAAGGGAATTCT-----------GAGGCTGCGTTTGTCAC
WT               GCCATGTTTGGAAGGGAATTCTGCTACGCTGTTGAGGCTGCGTTTGTCAC
                 **********************           *****************
```

zebrafish *tyr*

#1

cr1:Δ7-I3

```
1                GCCTTTAGTGTTTTACAACCAAACCTGCCATGG----GGAAACTACATGG
WT               GCCTTTAGTGTTTTACAACCAAACCTGCCAGTGCGCCGGAAACTACATGG
                 ******************************       *************
```

#2

cr1:Δ22

```
2                TTTAGTGTTTTACAACCAAACCT----------------------GGGGT
WT               TTTAGTGTTTTACAACCAAACCTGCCAGTGCGCCGGAAACTACATGGGGT
                 ***********************                      *****
```

#3

cr1:Δ12

```
3                TAGTGTTTTACAACCAAACCTGCCA------------CTACATGGGGTTG
WT               TAGTGTTTTACAACCAAACCTGCCAGTGCGCCGGAAACTACATGGGGTTG
                 *************************            *************
```

X. tropicalis slc45a2

#1

cr1:Δ8

```
1                CCCAGGAGTTTGTACAGTCTTGTCTGGCT--------CCATCTTGGGATT
WT               CCCAGGAGTTTGTACAGTCTTGTCTGGCTCATCAGCCCCATCTTGGGATT
```

#2

cr2:Δ14

```
2                GGCTATGTTTGGAAGGGAATTTTG--------------CAGCCTTCGTAA
WT               GGCTATGTTTGGAAGGGAATTTTGCTACGCTGTGGAGGCAGCCTTCGTAA
                 ************************              ************
```

#3
cr1:Δ7

```
3               CCCAGGAGTTTGTACAGTCTTGTCTGGCTC-------CCATCTTGGGATT
WT              CCCAGGAGTTTGTACAGTCTTGTCTGGCTCATCAGCCCCATCTTGGGATT
                ******************************       *************
```

#4
cr2:Δ9

```
4               ATGGCTATGTTTGGAAGGGAATTTTGCTACGCTGT---------CTTCGT
WT              ATGGCTATGTTTGGAAGGGAATTTTGCTACGCTGTGGAGGCAGCCTTCGT
                ***********************************         ******
```

X. tropicalis tyr

#1
cr2:Δ13

```
1               CTACTTGAATTTGGCTAAGCACACC-------------ATGTCATAGTCAC
WT              CTACTTGAATTTGGCTAAGCACACCACCAGCCGTGACTATGTCATAGTCAC
                *************************             *************
```

#2
cr2:Δ7

```
2               CTACTTGAATTTGGCTAAGCACACCACC-------ACTATGTCATAGTCAC
WT              CTACTTGAATTTGGCTAAGCACACCACCAGCCGTGACTATGTCATAGTCAC
                ****************************       ****************
```

#3
cr2:Δ7-I9

```
3               CTACTTGAATTTGGCTAAGCACACCACCAGTCATAGTCATATGTCATAGTC
WT              CTACTTGAATTTGGCTAAGCACACCACCAGCCGTGAC--TATGTCATAGTC
                ****************************** * *     ************
```

Supplementary Figure 4. Examples of mutations obtained with LbCpf1-crRNA RNP complexes targeting *slc45a2* and *tyr* genes in zebrafish and *X. tropicalis.*
Two 48 hpf zebrafish or stage 47 *X. tropicalis* embryos per condition were collected for DNA extraction (see Methods). Different targeting regions were PCR amplified (Supplementary Data 1), cloned into pCR2.1-TOPO (Invitrogen) and sequenced. cr: crRNA target site (Supplementary Data 1); Δ:deletion; I:insertion. CRISPR-Cpf1 target site is highlighted in green.

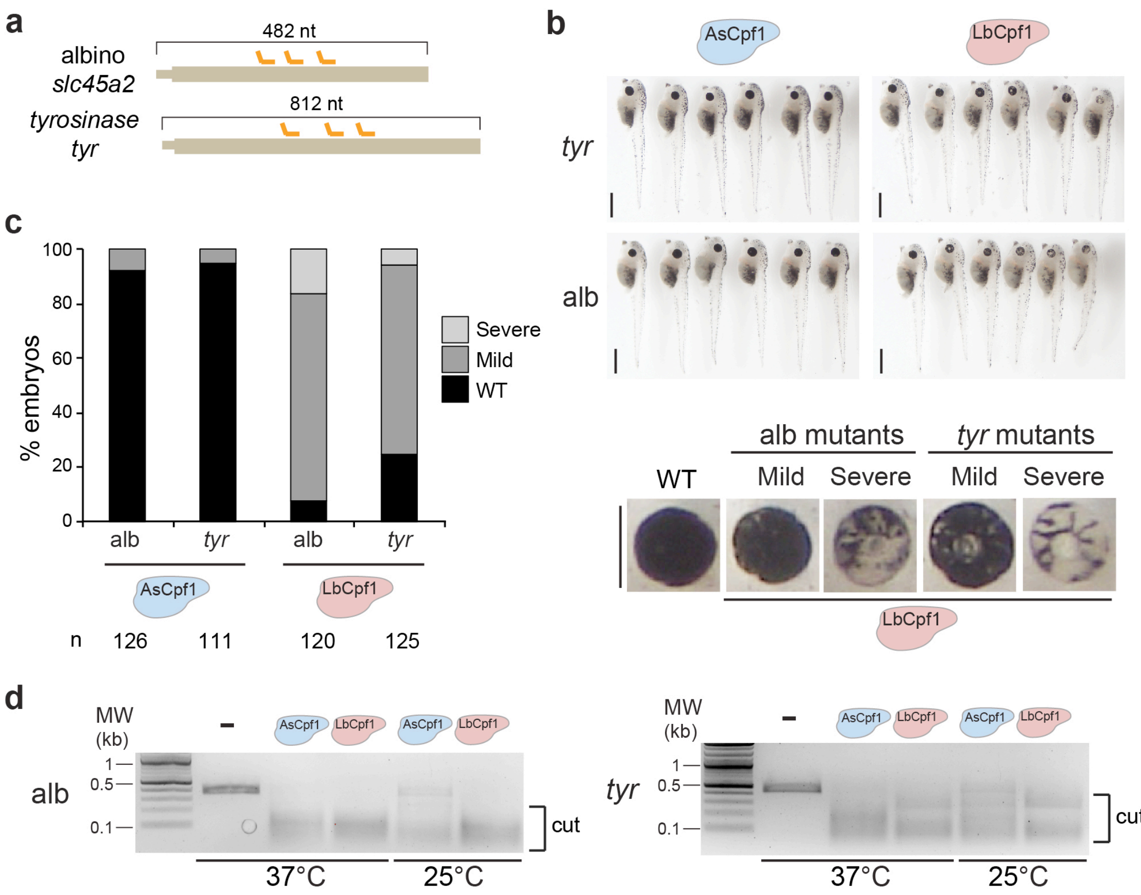

Supplementary Figure 5. LbCpf1-crRNA RNP complexes are an efficient genome editing system in *X. tropicalis*.
a. Diagram illustrating 3 crRNAs (orange) targeting *slc45a2* and *tyr* exon 1 in *X. tropicalis*.
b. Phenotypes obtained after injection of the AsCpf1-crRNA or LbCpf1-crRNA RNP complexes (20 fmol) containing a mix of 3 crRNAs targeting *slc45a2* (alb) or *tyr* in *X. tropicalis*. Lateral views (top; scale bar, 1 mm) and insets of the eyes (bottom; scale bar, 0.4 mm) of stage 47–48 embryos are shown.
c. Phenotypic evaluation of Cpf1-crRNA RNP complexes (20 fmol) injections targeting *slc45a2* (alb) or *tyr* in *X. tropicalis*. Stacked barplots showing the percentage of severe mutant (light gray), mild mutant (dark grey) and phenotypically WT (black) embryos at stage 47-48. Number of embryos evaluated (n) is shown for each condition.
d. In vitro cleavage assay using 30 pmol of AsCpf1 RNP or LbCpf1 RNP complexes containing a mix of 3 crRNAs (Supplementary Fig. 5a) and a ~380pb (~ 0.45 pmol) or ~440pb (~ 0.38 pmol*)* PCR product including crRNA target sites for *slc45a2* (*alb*) (left) and *tyr* (right), respectively. Incubations were carried out at 37°C and 25°C for 90 min. MW: Molecular weight marker (kilobase).

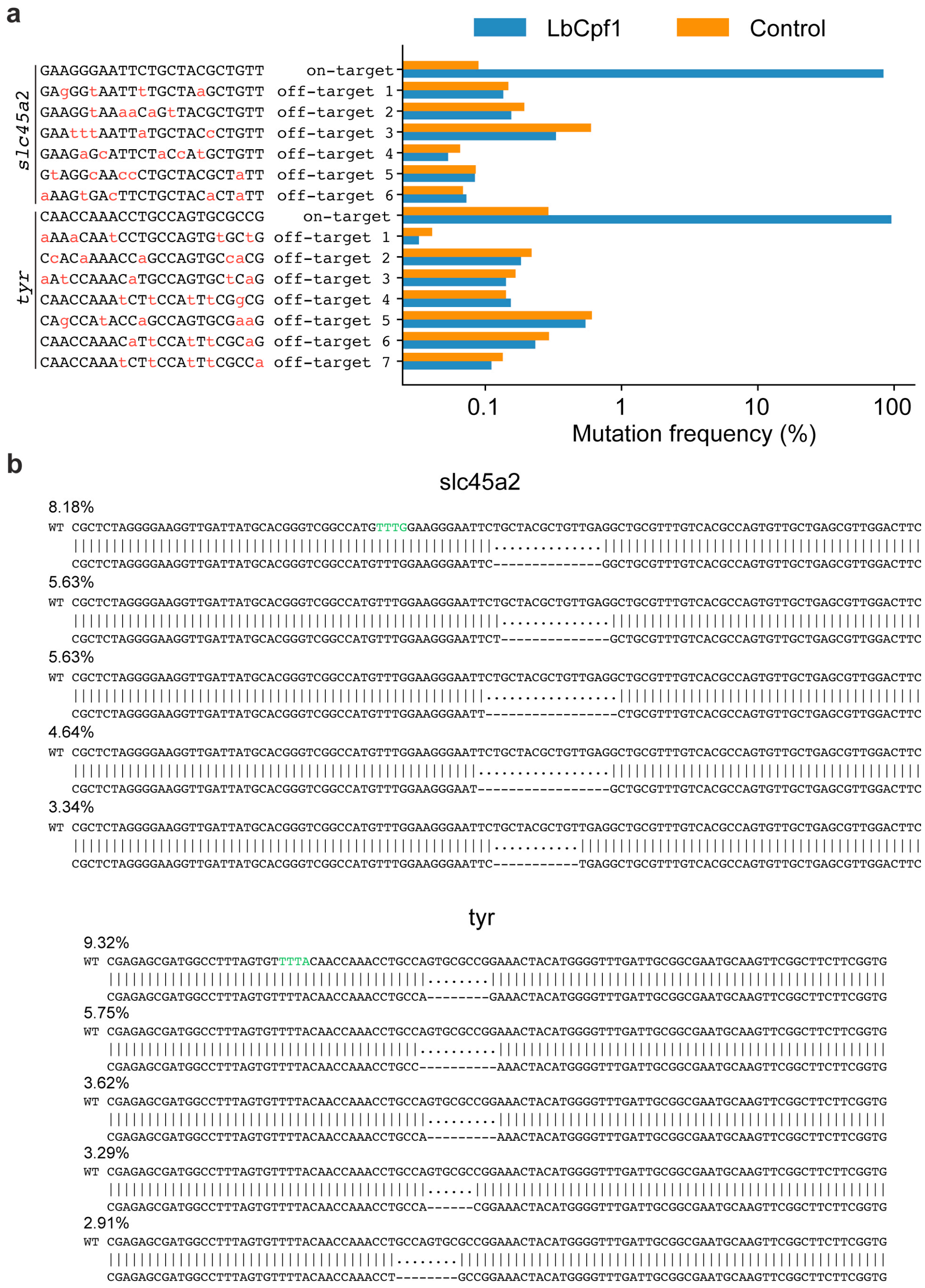

Supplementary Figure 6. Off-targeting analysis for highly efficient LbCpf1-crRNA RNP complexes.

a. Percentage (log-10 scale) of indel mutations (mutation frequency) in the potential off-target sites related to target alb 2 (*slc45a2*) and to target tyr 1 (*tyr*), including the on-target sites from injected (LbCpf1) or non-injected (Control). Mismatch bases in the potential off-targets in comparison to the target sites are shown in red and small case.

b. Examples of the most represented on-target indel mutations (% of total number of mutations per example is shown). WT PAM sequence is highlighted in green.

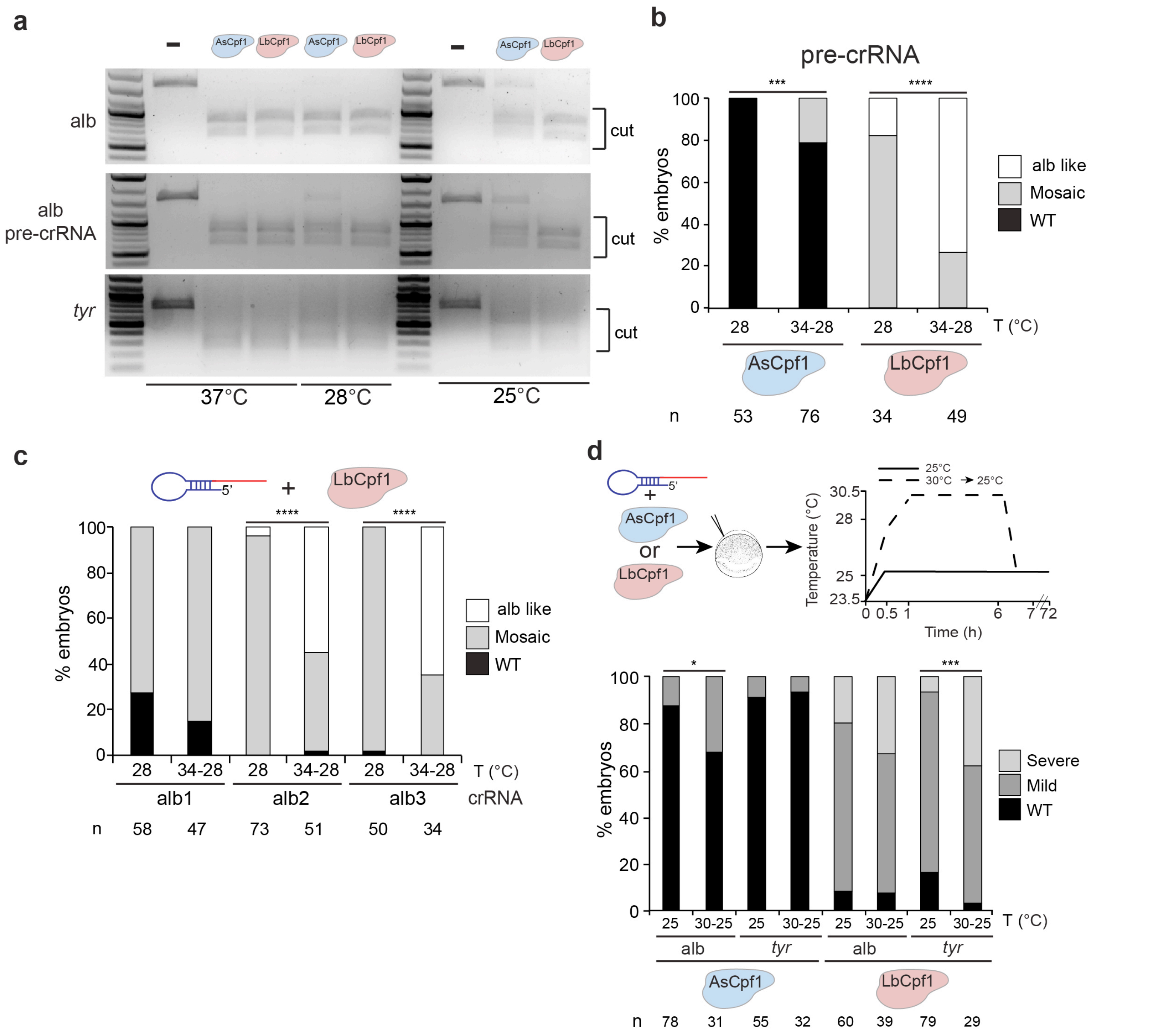

Supplementary Figure 7. Temperature is a key factor modulating Cpf1 activity *in vitro* and *in vivo*.

a. In vitro cleavage assay using AsCpf1 RNP or LbCpf1 RNP complexes containing a mix of 3 crRNAs (10 pmol) or pre-crRNAs (2.5 pmol) and a ~1.6 Kb (~ 0.11 pmol) PCR product (top and middle, respectively) containing crRNA target sites for *slc45a2* (alb) or a mix of 3 crRNAs (2.5 pmol) and a 0.77 Kb (~ 0.23 pmol*)* PCR product and containing crRNA target sites for *tyr*. Incubations were carried out at 37°C, 28°C or 25°C for 90 min. Molecular weight marker described in Supplementary Fig. 3c.

b. Phenotypic evaluation of LbCpf1-pre-crRNA RNP complexes (10 fmol) injections targeting *slc45a2* at different temperature incubations (T) (Fig. 2a). Stacked barplots showing the percentage of alb-like (white), mosaic mutants (grey) and phenotypically WT (black) embryos 48 hpf after injection. Number of embryos evaluated (n) is shown for each condition. χ^2 test (*** p< 0.001,**** p< 0.0001).

c. Phenotypic evaluation of individual LbCpf1-crRNA RNP complexes (10 fmol) injections targeting *slc45a2* at different temperature incubations (T) (Fig. 2a). Stacked barplots showing the percentage of alb-like (white), mosaic mutants (grey) and phenotypically WT (black) embryos 48 hpf after injection. Number of embryos evaluated (n) is shown for each condition. χ^2 test (**** p< 0.0001).

d. Schematic illustrating different temperature incubations after Cpf1-crRNA RNP complexes injections targeting *slc45a2* (alb) and *tyr* in *X. tropicalis* (top). Phenotypic evaluation of pre-crRNA-LbCpf1 RNP complexes (10 fmol) injections targeting *slc45a2 and tyr* at different temperature incubations (T) (bottom). Stacked barplots showing the percentage of severe mutant (light gray), mild mutant (dark grey)

and phenotypically WT (black) embryos at stage 47-48. Number of embryos evaluated (n) is shown for each condition. χ^2 test (*p< 0.05, ***p< 0.001).

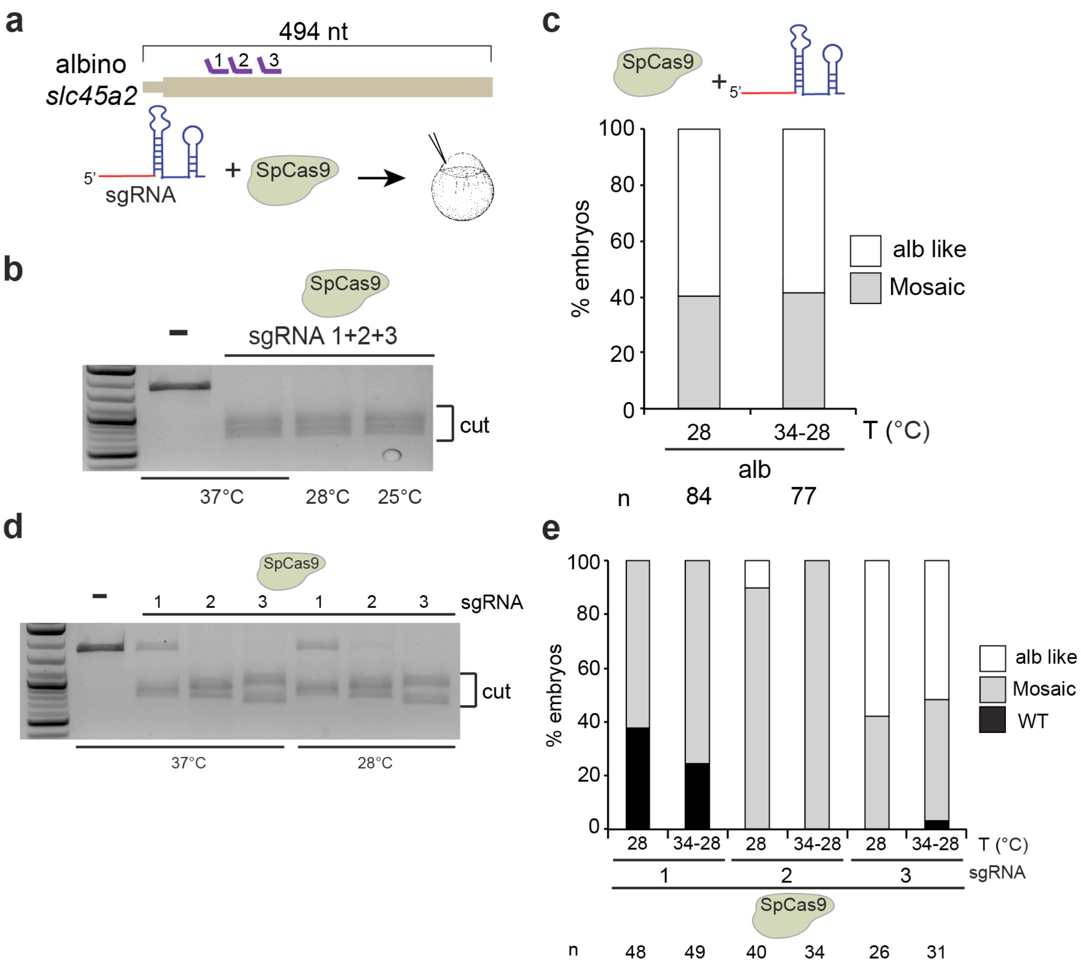

Supplementary Figure 8. SpCas9 activity is not modulated by temperature in zebrafish.

a. Diagram illustrating 3 sgRNAs (purple) targeting *slc45a2* exon 1 in zebrafish (top). Schematic showing sgRNAs (from above) that were assembled into SpCas9-sgRNA RNP complexes and injected into one-cell stage embryos (bottom).

b. In vitro cleavage assay using SpCas9-sgRNA RNP complexes containing a mix of 3 sgRNAs (10 pmol) and a ~1.6 Kb (~ 0.11 pmol) PCR product containing sgRNA target sites for *slc45a2*. Incubations were carried out at 37°C, 28°C or 25°C for 90 min. Molecular weight marker described in Supplementary Fig. 3c.

c. Phenotypic evaluation of SpCas9-sgRNA RNP complexes (10 fmol) injections targeting *slc45a2* at different temperature incubations (T) as described in Fig. 2a. Stacked barplots showing the percentage of alb-like (white) and mosaic (gray) embryos 48 hpf after injection. χ^2 test was performed and no significant differences were observed.

d. *In vitro* cleavage assay using 10 pmol of SpCas9-sgRNA RNP complexes containing individual sgRNAs and a ~1.6 Kb (~ 0.11 pmol) PCR product including sgRNA target sites for *slc45a2*. Incubations were carried out at 37°C for 90 min. Molecular weight marker described in Supplementary Fig. 3c.

e. Phenotypic evaluation of individual SpCas9-sgRNA RNP complexes (10 fmol) injections targeting slc45a2 at different temperature incubations (T) as described in Fig. 2a. Stacked barplots showing the percentage of alb-like (white), mosaic mutants (grey) and phenotypically WT (black) embryos 48 hpf after injection. Number of embryos evaluated (n) is shown for each condition. χ^2 test was performed and no significant differences were observed

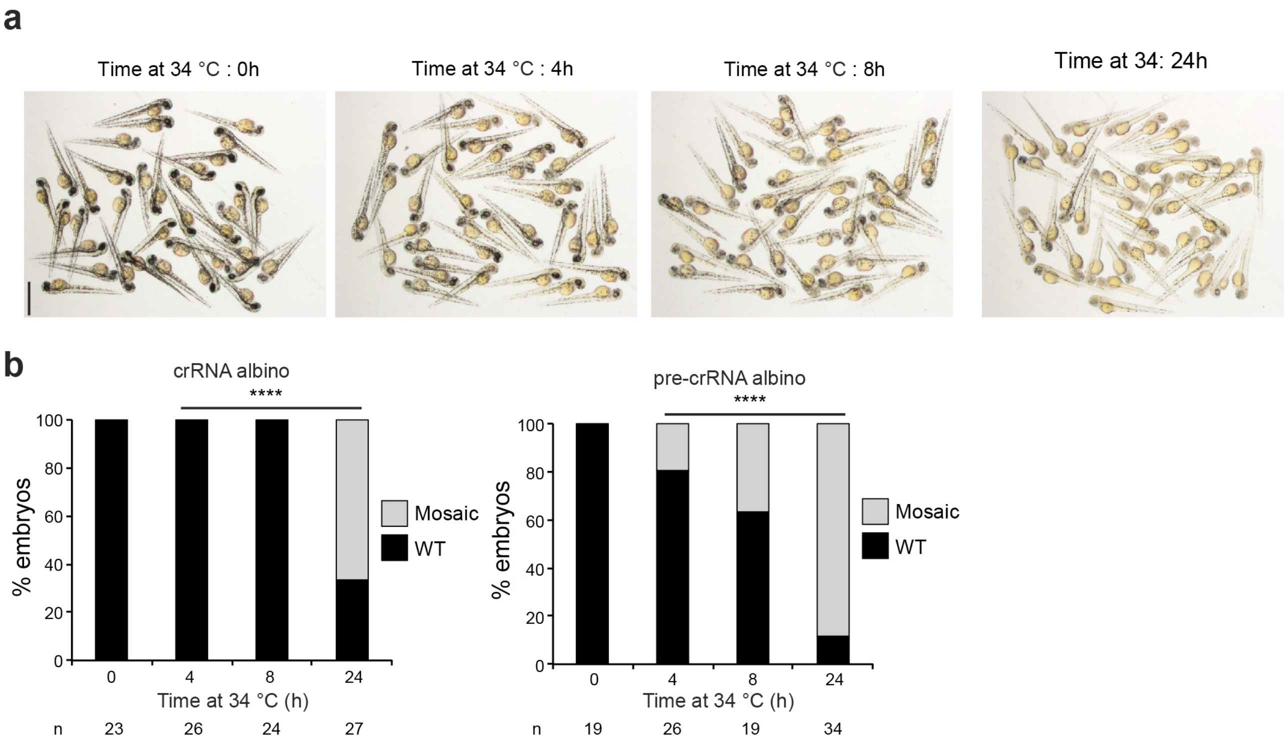

Supplementary Figure 9. Longer incubations at 34°C improve AsCpf1 activity *in vivo.*

a. A representative picture showing 48 hpf old embryos obtained after AsCpf1-crRNA RNP complexes injections targeting *tyr* in the conditions described in Fig. 2f. Scale bar, 1.25 mm.

b. Phenotypic evaluation of AsCpf1-crRNA (left) or AsCpf1-pre-crRNA (right) RNP complexes (10 fmol) injections targeting *slc45a2* (alb) in the conditions described in Fig. 2f. Stacked barplots showing the percentage of mosaic mutants (grey), and phenotypically WT (black) embryos 48 hpf after injection. Number of embryos evaluated (n) is shown for each condition. χ^2 test (****p< 0.0001).

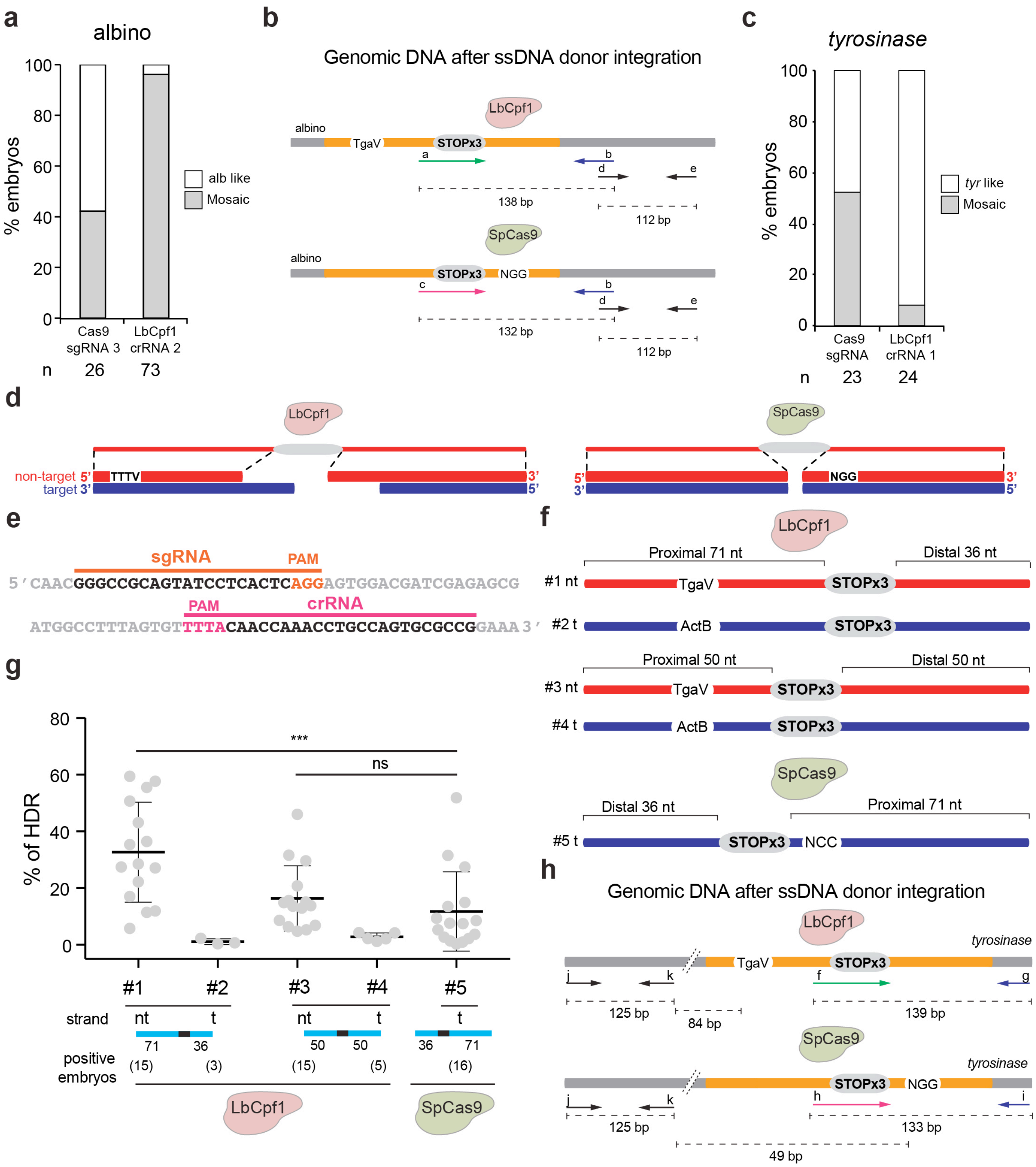

Supplementary Figure 10. LbCpf1-mediated homology-directed repair.

a. Phenotypic evaluation of SpCas9-sgRNA 3 (Fig. 4b, Supplementary Fig. 8e) and LbCpf1-crRNA 2 (Fig. 4b, Supplementary Fig. 3f) RNP complexes (10 fmol) injections targeting *slc45a2* and used in Fig 4. Stacked barplots showing the percentage of alb-like (white), mosaic mutants (grey) embryos 48 hpf after injection. Number of embryos evaluated (n) is shown for each condition.

b. Schema illustrating genomic DNA after ssDNA donor (containing 3 stop codons) integration in the albino locus. Specific primers (a,b,c) to amplify DNA integrations (orange) were used. Total DNA amount per embryo was calculated using primers (d,e) amplifying genomic DNA from the same locus (Supplementary Data 1, Methods).
c. Phenotypic evaluation of SpCas9-sgRNA and LbCpf1-crRNA 1 (Supplementary Data 1) RNP complexes (10 fmol) injections targeting *tyr and* used to quantify HDR. Stacked barplots showing the percentage of mosaic mutants (grey) and *tyr*-like (white) embryos 48 hpf after injection. Number of embryos evaluated (n) is shown for each condition.
d. Schematic illustrating ssDNA donor centered in the 3' end of the LbCpf1-crRNA double-strand break (left) or in the blunt end of the SpCas9-sgRNA double-strand break (right).
e. crRNA (pink line) and sgRNA (orange line) target sequences in the *tyrosinase* locus used for this analysis.
f. Schema illustrating different donor ssDNA (#1-#4) complementary to either the target strand (t) or non-target strand (nt) and with symmetric or asymmetric homology arms used in combination with LbCpf1-crRNA. PAM sequence was modified (TgaV/ActB) to prevent new editing post-HDR. An optimized ssDNA donor (#5) described for SpCas9-induced HDR[8] was used in combination with SpCas9-sgRNA RNP as reference for comparison (bottom).
g. qPCR quantification showing percentage of HDR from individual embryos when using LbCpf1 and different ssDNA donors in comparison with SpCas9. % of HDR: amount of integrated DNA per total amount of genomic DNA per embryo (see methods for details). Results are shown as the averages ± standard deviation of the means from 16 embryos in two independent experiments (n=8 embryos per experiment). Positive Embryos: number of embryos per condition showing a detectable qPCR amplification signal. The data were analyzed by Kruskal-Wallis test followed by Dunn's post-test for significance versus control condition (#5), (***) $P < 0.001$, (ns) not significant.
h. Schema illustrating genomic DNA after ssDNA donor (containing 3 stop codons) integration into the *tyrosinase* locus. Specific primers (f-i) to amplify DNA integrations (orange) were used. Total DNA amount per embryo was calculated using primers (j,k) amplifying genomic DNA from the same locus (Supplementary Data 1, Methods).

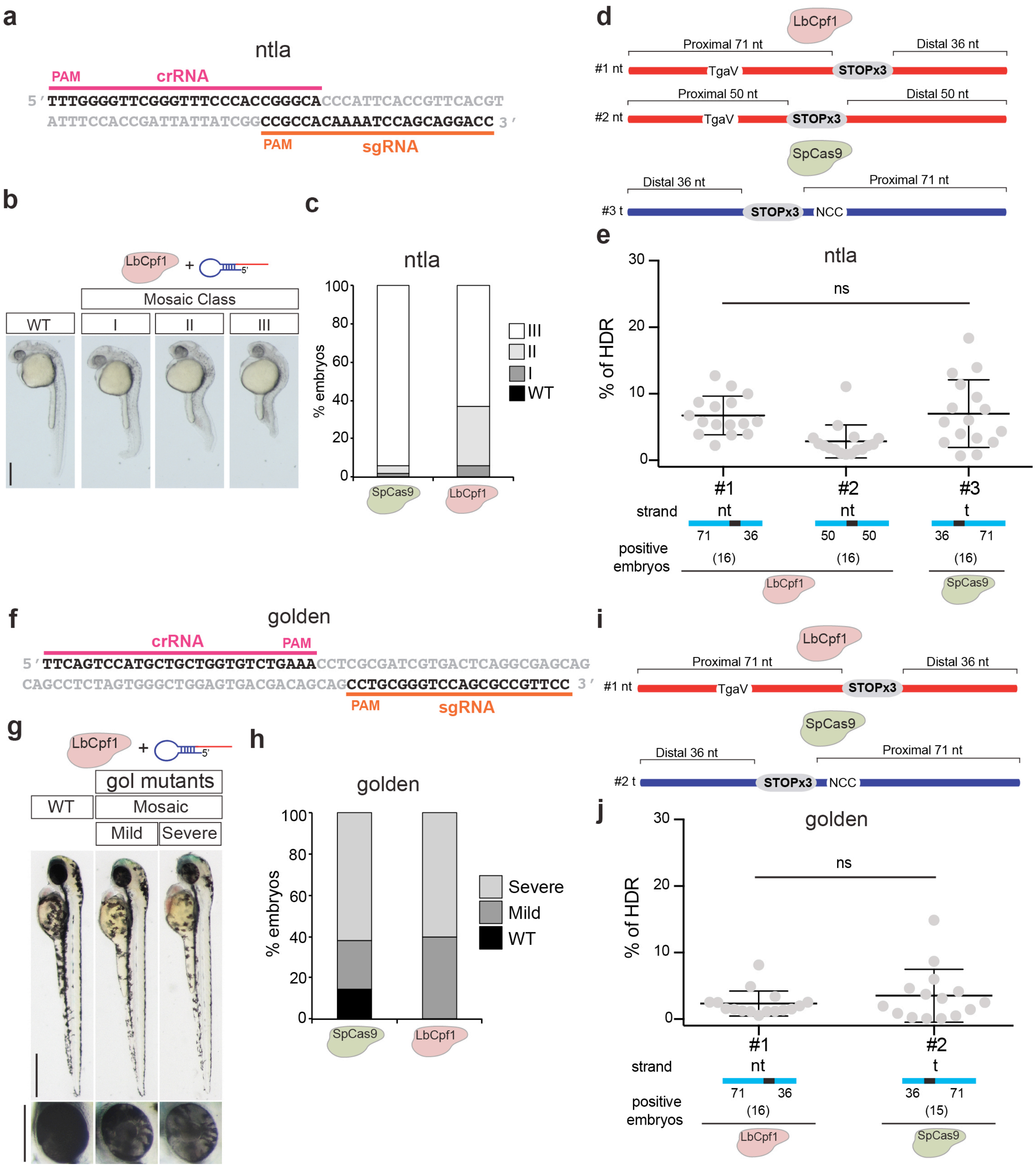

Supplementary Figure 11. LbCpf1-mediated homology-directed repair in ntla and golden loci.

a. crRNA (pink line) and sgRNA (orange line) target sequences in the *ta* (ntla*)* locus used for this analysis.

b. Phenotypes obtained after injection of the LbCpf1-crRNA and SpCas9-sgRNA RNP complexes (10 fmol) targeting ntla in zebrafish embryos (Lateral views). Levels of mosaicism compared to wild type (WT) were evaluated at 30 hpf. Class I: Short tail (least extreme). Class II: Absence of notochord and short tail (medium level). Class III: Absence of notochord and extremely short tail (most extreme). Scale bar, 0.36 mm.
c. Stacked barplots showing the percentage of different mosaic mutants described in b.
d. Schema illustrating different donor ssDNA (#1-#2) complementary to the target strand (t) and with symmetric or asymmetric homology arms used in combination with LbCpf1-crRNA. PAM sequence was modified (TgaV/ActB) to prevent new editing post-HDR. An optimized ssDNA donor (#3) described for SpCas9-induced HDR[8] was used in combination with SpCas9-sgRNA RNP as reference for comparison (bottom).
e. qPCR quantification showing percentage of HDR from individual embryos when using LbCpf1 and different ssDNA donors in comparison with SpCas9 in the conditions described in d. To measure % HDR, specific primers to amplify DNA integrations were used following a similar approach described in Supplementary Fig. 10b and h, and albino genomic primers were used to estimate genomic DNA amount (Supplementary Data 1, see Methods). % of HDR: amount of integrated DNA per total amount of genomic DNA per embryo (see methods for details). Results are shown as the averages ± standard deviation of the means from 16 embryos in two independent experiments (n=8 embryos per experiment). Embryos showing a PCR amplification signal detectable by qPCR were considered positive. The data were analyzed by Kruskal-Wallis test followed by Dunn's post-test for significance versus control condition (#3). No significant (ns) differences were found between LbCpf1 and SpCas9 in the optimized conditions (#1 vs #3).
f. crRNA (pink line) and sgRNA (orange line) target sequences in the *slc24a5* (golden) locus used for this analysis.
g. Phenotypes obtained after injection of the LbCpf1-crRNA and SpCas9-sgRNA RNP complexes (20 fmol) targeting golden in zebrafish embryos. Lateral views (top; scale bar, 0.5 mm) and insets of the eyes (bottom; scale bar, 0.25 mm) of the embryos at 48 hpf are shown.
h. Stacked barplots showing the percentage of different mosaic mutants described in g.
i. Schema illustrating a donor ssDNA (#1) complementary to the target strand (t) and with asymmetric homology arms used in combination with LbCpf1-crRNA. PAM sequence was modified (TgaV/ActB) to prevent new editing post-HDR. An optimized ssDNA donor (#2) described for SpCas9-induced HDR[8] was used in combination with SpCas9-sgRNA RNP as reference for comparison (bottom).
j. qPCR quantification showing percentage of HDR from individual embryos when using LbCpf1 in comparison with SpCas9 in the conditions described in i. To measure % HDR, specific primers to amplify DNA integrations were used following a similar approach described in Supplementary Fig. 10b and h and albino genomic primers were used to estimate genomic DNA amount (Supplementary Data 1, see Methods). % of HDR: amount of integrated DNA per total amount of genomic DNA per embryo (see methods for details). Results are shown as the averages ± standard deviation of the means from 16 embryos in two independent experiments (n=8 embryos per experiment). Positive Embryos: number of embryos per condition showing a detectable qPCR amplification signal. The data were subjected to unpaired two-tailed Mann-Whitney test and no significant differences (ns) were found between LbCpf1 and SpCas9.

Supplementary References

1. Mojica, F.J., Diez-Villasenor, C., Garcia-Martinez, J. & Almendros, C. Short motif sequences determine the targets of the prokaryotic CRISPR defence system. *Microbiology* **155**, 733-740 (2009).
2. Kleinstiver, B.P. et al. Engineered CRISPR-Cas9 nucleases with altered PAM specificities. *Nature* **523**, 481-485 (2015).
3. Esvelt, K.M. et al. Orthogonal Cas9 proteins for RNA-guided gene regulation and editing. *Nature methods* **10**, 1116-1121 (2013).
4. Ran, F.A. et al. In vivo genome editing using Staphylococcus aureus Cas9. *Nature* **520**, 186-191 (2015).
5. Hou, Z. et al. Efficient genome engineering in human pluripotent stem cells using Cas9 from Neisseria meningitidis. *Proceedings of the National Academy of Sciences of the United States of America* **110**, 15644-15649 (2013).
6. Zetsche, B. et al. Cpf1 is a single RNA-guided endonuclease of a class 2 CRISPR-Cas system. *Cell* **163**, 759-771 (2015).
7. Moreno-Mateos, M.A. et al. CRISPRscan: designing highly efficient sgRNAs for CRISPR-Cas9 targeting in vivo. *Nature methods* **12**, 982-988 (2015).
8. Richardson, C.D., Ray, G.J., DeWitt, M.A., Curie, G.L. & Corn, J.E. Enhancing homology-directed genome editing by catalytically active and inactive CRISPR-Cas9 using asymmetric donor DNA. *Nature biotechnology* **34**, 339-344 (2016).
