# Supplementary material for: CRISPR-Cpf1 mediates efficient homology-directed repair and temperature-controlled genome editing
Source: Nat Commun. 2017 Dec 8;8:2024. doi: 10.1038/s41467-017-01836-2 (PMC5722943; doi:10.1038/s41467-017-01836-2)
Supplement: Supplementary file 3 — Description of Additional Supplementary Files [file 41467_2017_1836_MOESM3_ESM.pdf]

Description of Additional Supplementary Files

File Name: Supplementary Data 1
Description: Sequences used in this study. Primers used to generate DNA IVT templates (crRNA or sgRNA).

File Name: Supplementary Data 2
Description: LbCpf1 off-target analysis. Target sequences analyzed and PCR primers used in Supplementary Fig. 6.

File Name: Supplementary Data 3
Description: txt file required to be used as is with the off-target analysis script provided in Methods.
